# Book Reviews

**Published:** 1800-01

**Authors:** 


*Observations on the Diseases of Seamen*, by G. BLANE, M. D. &c. the third edition, with corrections and additions, 8vo. pp. 650. London, Murray and Highley.

The former editions of this work, as well as the merit of the author, are too well known to medical readers, to require any thing more from us, than merely the announce of a new edition. We cannot, however, dismiss this third edition, without observing, that the corrections and additions are so important, as almost to entitle it to the expectations of a new work. Among these additions, we notice a chapter on ulcers, which Dr. B. believes are frequently contagious; and a chapter on casualties. Dr. B. approves of the nitrous fumigation, recommended by Dr. C. Smith, as a means of eradicating infection, in preference to his own fumigation with sulphur and cahrcoal.

The affusion of cold water in fevers is mentioned, but not on the author's own experience. In a word, Dr. B. appears to have neglected no sources of information, since the publication of the preceding edition.

---

*Medical Cases and Remarks*, Part I. *on the good Effects of Salivation in Jaundice, arising from Calculi.* Part II. *on the Free Use of Nitre in Hæmorrhages.* By T. GIBBONS, M. D. 8vo. pp. 100. London, Murray, &c.

Dr. G. has advanced twelve cases of jaundice, in support of his opinion respecting the solvent powers of calomel; from which it appears to be a valuable remedy in that disease. The usual formulæ are:

℞. Calomel. gr. j. Pulv. Rhei et Confect. Opiat. aa. gr. v. Syr. q. s. f. bolus bis die sumendus; or,

℞. Calomel. Pil. aloes et Confect. Opiat. aa. gr. iij. Syr. q. s. f. bolus mane & vespere sumendus; which are continued till the mouth becomes affected.

In the 2d Part, Dr. G. in cases of hæmorrhage gives Nitre in doses of ʒj. every four or five hours, with apparent success. Some opinions respecting the modus operandi are given by the Author, and his friend, Dr. Drake. The work concludes with some observations on the abuse of flannel waistcoats next the skin, which Dr. G. believes are generally worn too long without washing.

---

*Some Observations on the Bilious Fevers of* 1797, 1798, and 1799, by R. PEARSON, M. D. Physician to the General Hospital, near Birmingham, &c. 8vo. pp. 39. London. Seeley, &c.

In an advertisement prefixed to this pamphlet, Dr. P. says, "It has fallen to my lot to see the Fever described in the following pages, pages, throughout all its ftages, and under a vaft variety of forms, in this town and neighbourhood. Obferving fomething anomalous and peculiar in its character, I was induced to watch it with clofe attention. I here offer fuch facts relative to its hiftory and treatment, as my experience has hitherto furnifhed. They are but the prelude of a more complete fet of obfervations, which I hope to communicate hereafter. In the mean time, I flatter myfelf this fmall pamphlet will be of fome ufe, by calling forth the attention of Medical Practitioners to the true nature and proper treatment of that epidemic."

On the diftinction between this fever and typhus, Dr. P. obferves, " In its firft ftage, this fever did not appear to be contagious; but it was evidently fo after the eleventh or fourteenth day, when the typhoid ftate was induced. At this period it fpread, in many inftances, through whole families. The contagious nature of this fever has procured it very generally the name of Typhus; from which, however, it differs (1ft) in being accompanied, during its firft ftage, with little mufcular debility; (2dly) in being accompanied with a more tenfe pulfe; (3dly) with more violent head-ach, vomitings, and fenfibility of the ftomach; and (4thly) in being, for the moft part, of a remittent or intermittent type. Unlike typhus, it not only bears, but requires large evacuations upwards and downwards, and fome lofs of blood. A diarrhœa, fo frequently hurtful, and even mortal, in typhus, is, in this fever, for the moft part falutary."

The treatment recommended, confifts in V. S. or the ufe of leeches, during the firft three or four days in particular fubjects; emetics, purgatives of calomel, and neutral falts, or calomel joined with antimonial powder; faline febrifuges, with antimonial or ipecac. wine; opiates appeared to anfwer beft in clyfters, and thefe, as well as blifters, fucceeded better after the firft week, than during the firft five or fix days, unlefs when lethargic fymptoms appeared.

When the febrile fymptoms abated, bitters feemed to anfwer better than the bark. — Particular fymptoms required particular treatment; for an account of thefe, we refer to the work itfelf, which will well repay the perufal.

